# Prescription of Non-Occupational Post-Exposure HIV Prophylaxis by Emergency Physicians: An Analysis on Accuracy of Prescription and Compliance

**DOI:** 10.1371/journal.pone.0153021

**Published:** 2016-04-12

**Authors:** Stefano Malinverni, Agnès Libois, Anne-Françoise Gennotte, Cécile La Morté, Pierre Mols

**Affiliations:** 1 Department of Emergency Medicine, Centre Hospitalier Universitaire Saint Pierre, Université Libre de Bruxelles, Brussels, Belgium; 2 Infectious Diseases Department, Centre Hospitalier Universitaire Saint Pierre, Université Libre de Bruxelles, Brussels, Belgium; Mayo Clinic, UNITED STATES

## Abstract

We conducted a retrospective analysis of data from a prospective nPEP (non-Occupational Post Exposure Prophylaxis) registry based on patients consulting at one academic Emergency department located in Brussels, Belgium. We review here 1,357 cases consulting from January 2011 to December 2013.The objective of the study is to determine whether emergency physicians prescribe nPEP according to national guideline with support from IDS (infectious disease specialists). As this intervention has a high cost we wanted to verify correct allocation of treatment to high risk patients. Moreover we wanted to determine whether compliance to nPEP when prescribed by an Emergency Physician was different from literature reports. Finally we wanted to describe the population consulting for nPEP at our center. Emergency physicians prescribed nPEP more frequently in high risk exposures (98.6%) compared to intermediate risk exposures (53.2%); adequately allocating resources from a public health perspective. Appropriateness of prescription when evaluated according to nPEP Belgian guidelines was 98.8%.Compliance with nPEP prescribed by Emergency physicians was 60% in our study. Compliance was the highest in MSM (Men who have Sex with Men) while sexual assault victims showed the lowest compliance. Altogether this study suggests that Emergency physicians can safely and adequately prescribe nPEP when supported by a comprehensive guideline. Recognizing intrinsic differences within heterogeneous populations consulting for nPEP may improve compliance to this high-cost public health intervention.

## Introduction

HIV infection is not an instantaneous consequence of HIV exposure as it takes 48 to 72 hours for HIV virus to become detectable in regional lymph nodes and around 5 days to disseminate in the blood. Therefore a window of opportunity exists between exposure and infection [1]. PEP (Post-exposure prophylaxis) has been proven efficacious in animal models of HIV exposure [2] with success rates depending both on the interval between exposure and treatment and on the total treatment duration [3]. Data on humans, from a case–control study on health workers, showed that treatment with zidovudine after needle-stick exposures reduced risk of seroconversion for HIV by 81% [4]. Following those results nPEP (non-occupational post-exposure prophylaxis) was proposed as an intervention to reduce HIV transmission following an unexpected exposure, such as condom failure or sexual assault.

In Belgium 1115 new cases of HIV were diagnosed in 2013 [5]. A detailed guideline for the delivery of nPEP has been available in Belgium since 2009 [6] covering both sexual and non-sexual exposures (S1 Table). Detailed guidelines compiling all types of exposures may be beneficial for both clinicians and patients, favouring a better and more rational pattern of prescription. Belgian guidelines take into account different factors, such as type of exposure, risk factors associated with exposure, HIV status and viral load of the source person. If the HIV status is unknown, the prevalence of HIV in different subpopulations is used as a proxy. Belgian guidelines do not differ considerably from American [7] or UK ones [8] in terms of risk stratification. No recommendations in terms of drug regimen are made in the Belgian guidelines [6].

The main aim of our study is to inquire whether our prescription strategy, which relies on the joint work of EP (Emergency Physicians) and IDS (Infectious Disease Specialists) coupled with a detailed Guideline is a safe practice allowing for appropriate prescriptions of nPEP according to Belgian guidelines. The secondary objective is to verify whether completion rates for nPEP, when prescribed by EP, are in line with those reported in the literature. The tertiary objective is to describe the characteristics of the population and of exposure types leading to nPEP requests.

## Methods

### Study design and setting

We conducted a retrospective analysis of patients consulting for nPEP at Centre Hospitalier Universitaire Saint Pierre between the 1st January 2011 and the 31st December 2013. Data were extracted from a database of nPEP episodes filled in by EP in conjunction with IDS. An automated data extraction was performed by a statistician not involved in the study and all data were anonymized. A manual review of medical charts was done in cases of ambiguous data. The data included information about patient demographic characteristics, exposure type, source person profile, follow-up results such as haematochemistry and serology, reported side-effects, reported reasons for interruption of therapy and compliance as self-reported by the patient during follow up visits.

Additionally the drug regimen and the duration of treatment were extracted from both patients’ records and from two hospital pharmacy databases. In case of incongruities, data from medical chart were assumed as more reliable in all cases and used in the analysis. In cases of discrepancies between the two pharmacy databases an error of under reporting was assumed in all cases and the longer treatment duration was used in the analysis.

The setting of our study is Centre Hospitalier Universitaire Saint Pierre, an HIV reference hospital located in a relatively deprived and high immigration area of the centre of Brussels, Belgium. Seven other hospitals in Belgium dispense free of cost nPEP, however our centre alone provided more than 50% of national consultations for nPEP in 2011. The nPEP drugs, in our setting, are provided free of cost from the hospital pharmacy. Our Institution provides nPEP through a close collaboration between the Emergency Department and the STI (Sexually Transmitted Infection) clinic. Patients consulting for nPEP are seen in the Emergency Department by an EP available 24/7 on a protocol basis. An exposure history is recorded by the EP in a standardised electronic form, and blood samples for baseline serology are taken. The decision to start treatment is taken by the EP according to Belgian guidelines. An IDS is available by phone 24/7 to assist the EP in case of doubts. Whenever the sources’ HIV status is unknown, the patient is encouraged to contact his or her partner in order to undergo an HIV test. Depending on the outcome of the risk assessment, a five-day starter pack is provided. Starter packs consists of a fixed drug regimen of lamivudine, stavudine and ritonavir-boosted lopinavir. Risk-reduction and medication adherence counselling are offered to all patients and an appointment at the STI clinic is given within 5 days. This follow-up visit is carried out in the STI clinic by an IDS, where results of baseline serology are interpreted, source person-HIV test results are re-evaluated if available and the risk of exposure is re-assessed. Prolongation of treatment beyond 5 days, adaptation of the drug regimen and additional follow-up visits are decided by the IDS on a case-by-case basis. In exceptional cases antiretroviral drugs are tailored at the start of the treatment according to known resistances.

Ethical approval for this study was obtained by the Ethical Board of Centre Hospitalier Universitaire Saint Pierre. No informed consent was asked from study participants as the data were analysed anonymously and no intervention involved study participants.

### Outcome Measures

Appropriateness of prescriptions and strength of the indication to treat was evaluated according to nPEP national guidelines [6].

A prescription for nPEP was considered appropriate if all the following conditions were fulfilled:

1. Delay between exposure and consultation was less than 72 hours.
2. No condom was used or, if condom was used, it broke or slid during intercourse.
3. Exposure type was considered at risk according to Belgian guidelines (S1 Table).

Strength of treatment recommendation was evaluated according to Belgian guidelines:

1. A “recommend treatment” statement was considered a strong indication to treat given the high-risk exposure (S1 Table).
2. A “consider treatment” statement was considered a possible indication to treat given the intermediate risk. Decision whether to treat was left to the clinician in charge according to his/her clinical judgment (S1 Table).
3. A “discourage treatment” statement was considered a strong indication against treatment given the low risk of exposure (S1 Table).

Patients returning for nPEP consultations following a new episode of exposure were considered as having had separate episodes. Recurrence was therefore defined as consulting twice or more during the study period for separate episodes.

Exposed persons receiving 28 days of treatment were considered compliant. Compliance was measured from pharmacy records and from medical records. Patients self-reporting having completed the full treatment course during a follow up visit more than 4 weeks after exposure were considered compliant. Patients having received at least 28 days of treatment from the pharmacy were considered compliant unless reported otherwise by medical charts. Our pharmacy does not dispense the whole treatment after the first follow-up visit. It only dispenses treatment up until the next follow-up visit, reducing therefore the possibility of errors driven by patients retrieving the whole treatment from pharmacy after the first consultation without taking it. Patients receiving more than 1 but less than 28 days of treatment were considered as having received an incomplete treatment course and therefore incompliant, unless treatment was interrupted by a IDS. Whenever treatment was interrupted by IDS, patients were not considered in the compliance analysis.

Data are presented with descriptive statistics. All data were analysed using STATA 11. Missing data were considered as missing at random and only available data were used for the analysis.

## Results

### Characteristics of the patients

During the 3 year period of analysis, 1357 consultations for nPEP were analysed.

Sexual exposures represented most of the accidental exposures, leading to 1302 consultations (96%). Non-sexual exposures such as bites, exposure to abandoned needles and needle sharing practices, accounted for the remaining 55 consultations (4%). Of the latter, only one case of needle sharing was identified.

Table 1 shows the patients’ characteristics. Most of them were male, 71.7%, with a mean age of 31.5 years. Sexual orientation of the exposed persons consulting for nPEP was MSM (men who have sex with men) in 36.8% of cases, heterosexual in 58.0% and bisexual in 5.2%. Fifty-three per cent of persons weren’t of Belgian nationality. 76% of consulting patients were covered by health insurance. 11.9% consultations were from persons consulting more than once during the study period. 15.4% of exposed persons were victims of sexual assault, out of which 91% were heterosexual women.

**Table 1:** Patient baseline characteristics.

Patient baseline characteristics								
	All		Heterosexual men		Heterosexual women		MSM and Bisexuals	
	mean	sd	mean	sd	mean	sd	mean	sd
Age (years)	31.5	9.5	31.7	9.1	28.4	9.6	33.4	9.1
	%	n	%	n	%	n	%	n
Male	71.7	973	100	412	0.0	0	98.4	561
Sexual exposures	96	1302	96.6	398	96.5	362	95.1	542
MSM	36.8	499	0.0	0	0.0	0	87.5	499
Bisexual	5.2	71	0.0	0	0.0	0	12.5	71
Heterosexual	58.0	787	100.0	412	100.0	375	0.0	0
Non Belgian	52.7	716	52.9	218	58.9	221	48.6	277
Health insurance	76	1031	82.5	340	71.5	268	74.2	423
Multiple nPEP demands	11.9	162	10.7	44	6.1	23	16.7	95
Sexual assault victim	15.4	208	0.0	0	50.7	190	3.2	18
Compliance	60.2	522	65.2	146	43.2	98	66.8	278

Among sexual exposures, as described in Table 2, unprotected receptive anal intercourses (URAI) represented 25% of cases, unprotected insertive anal intercourse (UIAI) accounted for 19.5% and unprotected vaginal receptive intercourse (URVI) 25.8%. More than one type of exposure was present in 32.2% of cases.

**Table 2:** Sexual exposure characteristics by episode.

Sexual exposure cheracteristics by episode								
	All		Heterosexual men		Heterosexual female		MSM and bisexual	
	%	n	%	n	%	n	%	n
Insertive anal	19.5	265	11.4	47	0.0	0	38.2	218
Receptive anal	25.0	339	0.0	0	16.0	60	48.9	279
Vaginal insertive	25.9	352	83.3	343	0.0	0	1.6	9
Vaginal receptive	25.8	350	0.0	0	91.5	343	1.2	7
Oral insertive	19.7	268	27.2	112	0.0	0	27.4	156
Oral receptive	22.2	301	0.7	3	25.6	96	35.4	202
Multiple exposures	32.2	437	26.9	111	30.1	113	37.4	213
Mucosal lesions	13.9	189	11.4	47	10.4	39	18.1	103
Multiple partners	8.3	113	3.9	16	10.7	40	10.0	57

As shown in Table 3 16.7% of source persons were known to be HIV infected.

**Table 3:** Source person characteristics.

Source patient carachteristics								
	All		Heterosexual male		Heterosexual female		MSM and Bisexual	
	%	n	%	n	%	n	%	n
Male	68.1	924	0.5	2	99.5	373	96.3	549
MSM	40.8	553	0.5	2	1.3	5	95.8	546
High prevalence country	16.1	219	23.5	97	18.7	70	9.1	52
HIV+	16.7	226	12.6	52	7.7	29	25.4	145
IDU	4.1	56	3.6	15	6.9	26	2.6	15
Commercial sex worker	13.9	189	34.5	142	4.5	17	5.3	30
Concurrency	25.1	341	25.5	105	17.3	65	30.0	171
Former prisoner	0.9	12	0.0	0	2.7	10	0.4	2

In the remaining cases, when HIV status was unknown, risk factors for HIV were frequent. In 36.3% of cases the source person was a MSM, in 4.3% the source was an intra-venous drug-user (IDU), in 17% a commercial sex worker and in 1.1% a former prisoner. 29.3% of source persons were engaged in multiple relationships. In 18.3% of the episodes the source person was an alleged sexual aggressor and in 19.6% of all episodes the source person was from a country with more than 2% prevalence of HIV e.g. nations within Sub Saharan Africa, South East Asia, former Soviet Union and the Caribbean. One or more of the aforementioned risk factors was present for 80% of the consultations resulting in nPEP prescription in which the source person had an unknown HIV status as shown in Table 4.

**Table 4:** Source person characteristics when HIV status is unknown.

**Source person characteristics when HIV is unknown n = 1020**		
** **	**%**	n
MSM	36.4	371
IDU	4.3	44
Commercial sex worker	17.1	174
Participate to orgies	4.3	44
Concurrency	29.4	300
Former prisoner	1.1	11
Alleged Sexual aggressor	18.3	187
High-prevalence country	19.6	200
At least one risk factor	56.0	571
**HIV status is unknown and nPEP prescribed n = 716**			
At least one risk factor	80.0	571

### Main results

**Fig 1:**
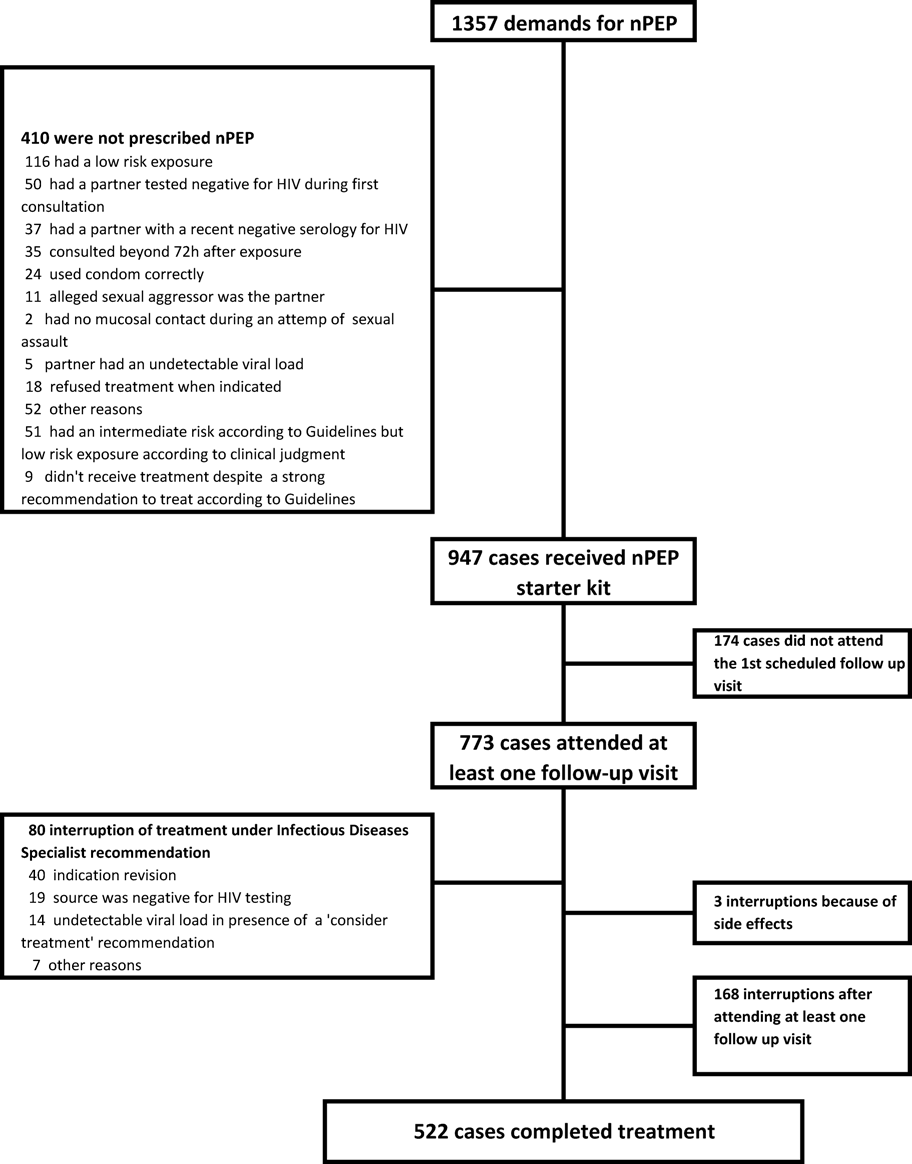
Study flow diagram.

Out of the 1357 demands analysed, nPEP was prescribed in 947 (69%) cases. In 410 of these initial nPEP demands an nPEP regimen was not prescribed. A clear contraindication to treatment initiation, such as a delay of more than 72h, partner’s recent negative serology, negative HIV testing of the source, a refusal of an indicated treatment or other could be identified in 350 (85%) cases. Detailed information about why patients were not prescribed nPEP is shown in Fig 1. According to Belgian guidelines, the transmission risk was intermediate in 51 of the remaining 60 episodes. A nPEP was not prescribed in these cases based on the clinical judgment of the EP, often guided by on-call IDS, who considered the exposure to be of low risk. Among the remaining 9 episodes for which nPEP was not prescribed, the risk of infection was nevertheless high according to Belgian guidelines, meaning that those patients had a theoretically strong recommendation for treatment. Moreover, 7 patients with a clear contraindication to treatment were put on treatment. In total a prescription error occurred in 1.2% of all consultations.

Altogether nPEP was prescribed in 98.6% (679/688) of patients consulting with a strong indication to treatment. Patients with an intermediate indication for treatment received nPEP in 52.8% (261/494) of risk exposures. Finally nPEP was prescribed in 4% (7/175) of patients with a clear contraindication to treatment.

Out of the 947 cases for which a therapy was started, treatment was subsequently withdrawn during follow-up visits in 80 cases (8%) according to medical advice from an IDS (Fig 1). Treatment was halted in 40 patients following an indication revision by the IDS: in 19 cases because a late HIV test on the source person was negative, and in 14 cases because of the confirmation of an undetectable viral load of the source together with a low risk exposure. In 7 cases, other reasons were listed.

Out of the 867 episodes for which a full 28 day nPEP regimen was advised, 522 (60%) completed treatment without interruptions while 174 (20%) didn’t attend the first scheduled follow-up visit. 177 patients (20%) experienced some form of side effects during treatment and three patients interrupted treatment because of side effects. Compliance in sexual-assault victims was lower than with other exposures with only 40% completing the 28 days regimen.

Six patients tested positive at initial HIV screening test during the first consultation for nPEP. During the study period we observed one episode of seroconversion at four months post-exposure in a MSM patient having had URAI with a HIV positive source and having received a complete nPEP regimen without reporting successive at risk behaviours, which represents a probable PEP failure.

## Discussion

Our study describes the results from three-years of nPEP prescription practice in Centre Hospitalier Universitaire Saint Pierre. Our results show encouraging data on the accuracy of nPEP prescription by EP. 1357 consultations resulted in 947 nPEP prescriptions. Among the 410 consultations with no prescribed treatment a clear contraindication was present in 350 cases. In 51 remaining cases risk was intermediate and patients were refused treatment on a case-by-case evaluation according to clinical judgment of both the EP and the IDS. In 9 cases, despite a high-risk exposure and strong recommendation to treat according to Belgian guidelines, treatment was not provided which represents a medical error by the EP. In 7 cases, treatment was prescribed despite a clear contraindication representing an over prescription error by the EP. Altogether this adds up to a prescription error in 1.2% of all consultations. Given that in 98.6% of cases with a strong recommendation to nPEP patient did receive treatment and in only 0.5% of total cases treatment was prescribed against national guidelines, we consider that treatment allocation by the EP was adequate. Part of this result can be explained by the opportunity to consult an IDS in case of doubts. Another factor explaining this high prescription accuracy might be the availability of a comprehensive guideline.

Some of the inappropriate prescriptions of nPEP may be due to inexperienced clinicians facing frightened patients consulting with a clear and un-negotiable plea for nPEP. In such cases, EP might be inclined to a more conservative and legally protective attitude, resulting in prescribing nPEP against National guidelines. Offering better training to EP about nPEP prescription and better defining the legal frame of nPEP might allow an even better adequacy of prescription.

Overall, our analyses show how nPEP can be effectively and safely prescribed by EPs if guided by clear guidelines and by the opportunity of discussing dubious cases with an IDS. Most of nPEP prescriptions were in cases of high risk exposures (72%) which benefited the most both from an individual and public health perspective [9]. This result suggests that EP correctly identified the patients with high-risk exposures.

Adherence to treatment in our series remains disappointingly low (60%), in agreement with a meta-analysis recently published [10]. Adherence was even lower in sex assault victims, 40%, a result slightly inferior to average compliance for developed countries [11]. Pill burden and side effects might partially explain these results [12]. In our series 19% of exposed persons who were prescribed nPEP experienced mild side effects and 3 had to interrupt treatment because of major side effects. Nevertheless this might be an underestimation of the problem given that patients might have interrupted treatment without attending a follow up visit because of side effects. Starter packs at our institution consist in most cases of lamivudine, stavudine and ritonavir-boosted lopinavir. Currently there are no guidelines for the choice of nPEP drug regimen in Belgium [6]. Our choice is a compromise between budgetary constraints and short term side effects profile, that keeps nPEP accessible to all patients without payment. Newer regimens with better tolerated drugs such as tenofovir and emtricitabine plus raltegravir as recommended by New York state Department of Health [13] might improve compliance whenever these drugs are available to all patients free of cost. Other factors leading to low compliance might be the out-of-pocket expenses related to follow-up visits, mental health problems [14], stigma associated to nPEP [15], alcohol use, illicit drug use [16], or lacking a health care insurance [17]. Psychological trauma after rape might partially explain the lower completion rate in this particular subgroup [18]. Another element contributing to the low compliance might be the provision of starter packs as this has been shown to be associated with lower completion rates [19]. Despite all of our patients having at least one scheduled counselling appointment, 174 (18.3%) never came to any follow-up visits and overall completion rates remained low.

Our retrospective analysis highlighted that most persons consulted because of accidental sexual exposures and that non-sexual exposure was a rare complaint (4%).

Subgroup descriptive analysis according to sexual orientation showed differences in type of exposure, compliance and recurrence in line with literature results [11] as it can be inferred from Tables 1–3. MSM tend to have a better compliance to nPEP but a higher tendency to recurrent risk exposures. Heterosexual men showed an intermediate compliance and frequent exposures with commercial sex workers. Heterosexual women consulted frequently following episodes of sexual assaults and showed the lowest compliance to treatment. Acknowledging those differences might help to better design socio-behavioural interventions in future, tailoring counselling programs for improving medication-adherence towards victims of sexual assault and counselling programs for risk-reduction behaviours in MSM. Following these results our centre changed the regimen for sexual assault victims into tenofovir and emtricitabine plus elvitegravir combined in one pill as a measure to simplify drug regimen and improve compliance.

80 nPEP treatments could safely be stopped early following risk revision by IDS. Early follow-up visits can be used as an opportunity to re-evaluate adequacy of nPEP prescription, which would lower costs for nPEP drugs and follow-up visits.

Six patients were diagnosed an HIV infection at the first consultation, highlighting the role of nPEP consultations as a screening tool in populations at risk.

Our study has both strengths and limitations. Strengths are the large number of cases reviewed, detailed information about exposed persons, source persons and exposures, the accurate record of pill prescription allowing an accurate estimation of compliance. Moreover it describes accurately the differences in behaviour and exposures of different subgroups. Additionally it describes the accuracy and safety of nPEP prescription when done by EP guided by IDS and a detailed guideline.

One of our weaknesses is the presence of missing data as not every file was accurately recorded. The second weakness lies in the fact that the study was carried out in a single healthcare centre. Our data are related to a particular healthcare system with free access to nPEP medications and to a particular population. Our results might therefore not apply to all other setting even though they might inspire future nPEP delivery systems.

## Conclusion

Retrospective analysis of a three year experience of our Emergency department attached to a tertiary care HIV centre shows that nPEP can be safely and correctly prescribed by an Emergency Physician if clear guidelines are provided and guidance by an Infectious Disease Specialist is available 24/7. Accuracy in prescription was 98.8%. Prescription of nPEP was done in high risk exposures in the majority of cases reflecting an effort towards a more efficacious and sustainable practice from a single patient and Public Health perspective. Moreover, we observed no differences in compliance rates when first nPEP contact is through an Emergency Physician compared to those reported in larger meta-analysis.

## Supporting Information

S1 Checklist STROBE checklist. (DOC)

S1 Dataset Study dataset. (XLSX)

S1 Table Belgian guideline table for nPEP risk assesment. High risk groups: MSM, IDU, commercial sex workers, multiple sexual partners, former prisoner High risk regions: Sub Saharan Africa, South America, South East Asia, former Soviet Union and Caribbean
1: syringe, spoon, filter, cotton, preparation, rinsing water
2: fresh blood visible on syringe, deep wound
Exposures at risk according to Belgian Guidelines are exposures for which a treatment is either recommended either considered
(PDF)

S2 Table Missing data for each variable of interest. (PDF)
