# Supplementary material for: Prescription of Non-Occupational Post-Exposure HIV Prophylaxis by Emergency Physicians: An Analysis on Accuracy of Prescription and Compliance
Source: PLoS One. 2016 Apr 12;11(4):e0153021. doi: 10.1371/journal.pone.0153021 (PMC4829160; doi:10.1371/journal.pone.0153021)
Supplement: S1 Checklist — (DOC) [file pone.0153021.s001.doc]

STROBE Statement—Checklist of items that should be included in reports of ***cross-sectional studies***

|  | Item No | Recommendation |
| --- | --- | --- |
| **Title and abstract** | 1 | (*a*) Indicate the study’s design with a commonly used term in the title or the abstract Page n2 and n4 |
|  |  | (*b*) Provide in the abstract an informative and balanced summary of what was done and what was found Page n2 |
| Introduction | | |
| Background/rationale | 2 | Explain the scientific background and rationale for the investigation being reported Page n3 |
| Objectives | 3 | State specific objectives, including any prespecified hypotheses Page n3 and n4 |
| Methods | | |
| Study design | 4 | Present key elements of study design early in the paper Page n4 |
| Setting | 5 | Describe the setting, locations, and relevant dates, including periods of recruitment, exposure, follow-up, and data collection Page n4 |
| Participants | 6 | (*a*) Give the eligibility criteria, and the sources and methods of selection of participants Page n4 |
| Variables | 7 | Clearly define all outcomes, exposures, predictors, potential confounders, and effect modifiers. Give diagnostic criteria, if applicable Page n5 and n6 |
| Data sources/ measurement | 8* | For each variable of interest, give sources of data and details of methods of assessment (measurement). Describe comparability of assessment methods if there is more than one group Page n5 and n6 |
| Bias | 9 | Describe any efforts to address potential sources of bias Page n5-6 |
| Study size | 10 | Explain how the study size was arrived at Page n4 |
| Quantitative variables | 11 | Explain how quantitative variables were handled in the analyses. If applicable, describe which groupings were chosen and why Page n6 |
| Statistical methods | 12 | (*a*) Describe all statistical methods, including those used to control for confounding Page n6 |
|  |  | (*b*) Describe any methods used to examine subgroups and interactions Not applicable |
|  |  | (*c*) Explain how missing data were addressed Page n6 |
|  |  | (*d*) If applicable, describe analytical methods taking account of sampling strategy not applicable |
|  |  | (*e*) Describe any sensitivity analyses not applicable |
| Results | | |
| Participants | 13* | (a) Report numbers of individuals at each stage of study—eg numbers potentially eligible, examined for eligibility, confirmed eligible, included in the study, completing follow-up, and analysed Page 9 and n10 + Fig1. |
|  |  | (b) Give reasons for non-participation at each stage Not applicable |
|  |  | (c) Consider use of a flow diagram Fig. 1 |
| Descriptive data | 14* | (a) Give characteristics of study participants (eg demographic, clinical, social) and information on exposures and potential confounders Pages 6-9 + table 1-2-3 |
|  |  | (b) Indicate number of participants with missing data for each variable of interest Supporting information “Missing data for each variable of interest” |
| Outcome data | 15* | Report numbers of outcome events or summary measures Page n9 and n10 |
| Main results | 16 | (*a*) Give unadjusted estimates and, if applicable, confounder-adjusted estimates and their precision (eg, 95% confidence interval). Make clear which confounders were adjusted for and why they were included Not applicable |
|  |  | (*b*) Report category boundaries when continuous variables were categorized Not applicable |
|  |  | (*c*) If relevant, consider translating estimates of relative risk into absolute risk for a meaningful time period Not applicable |
| Other analyses | 17 | Report other analyses done—eg analyses of subgroups and interactions, and sensitivity analyses Not applicable |
| Discussion | | |
| Key results | 18 | Summarise key results with reference to study objectives Page 9 and n10 |
| Limitations | 19 | Discuss limitations of the study, taking into account sources of potential bias or imprecision. Discuss both direction and magnitude of any potential bias Page 12 |
| Interpretation | 20 | Give a cautious overall interpretation of results considering objectives, limitations, multiplicity of analyses, results from similar studies, and other relevant evidence Page 10-12 |
| Generalisability | 21 | Discuss the generalisability (external validity) of the study results Page 12 |
| Other information | | |
| Funding | 22 | Give the source of funding and the role of the funders for the present study and, if applicable, for the original study on which the present article is based not applicable |

*Give information separately for exposed and unexposed groups.

**Note:** An Explanation and Elaboration article discusses each checklist item and gives methodological background and published examples of transparent reporting. The STROBE checklist is best used in conjunction with this article (freely available on the Web sites of PLoS Medicine at http://www.plosmedicine.org/, Annals of Internal Medicine at http://www.annals.org/, and Epidemiology at http://www.epidem.com/). Information on the STROBE Initiative is available at www.strobe-statement.org.
