# Supplementary material for: Prescription of Non-Occupational Post-Exposure HIV Prophylaxis by Emergency Physicians: An Analysis on Accuracy of Prescription and Compliance
Source: PLoS One. 2016 Apr 12;11(4):e0153021. doi: 10.1371/journal.pone.0153021 (PMC4829160; doi:10.1371/journal.pone.0153021)
Supplement: S1 Table — High risk groups: MSM, IDU, commercial sex workers, multiple sexual partners, former prisoner High risk regions: Sub Saharan Africa, South America, South East Asia, former Soviet Union and Caribbean 1: syringe, spoon, filter, cotton, preparation, rinsing water 2: fresh blood visible on syringe, deep wound Exposures at risk according to Belgian Guidelines are exposures for which a treatment is either recommended either considered (PDF) [file pone.0153021.s003.pdf]

Source patient characteristics	Type of sexual exposure						
	Anal receptive	Vaginal receptive	Anal or vaginal insertive	Oral receptive with ejaculation	Oral receptive without ejaculation	Oral insertive	Cunnilingus
**HIV positive**, detectable or unknown viral load	Recommended	Recommended	Recommended	Recommended	Consider	Discourage	Discourage
**HIV positive**, undetectable viral load (3 months)	Consider	Consider	Consider	Consider	Discourage	Discourage	Discourage
**HIV unknown**, high risk group or high risk region. Presence of mucosal risk factors	Recommended	Recommended	Recommended	Recommended	Consider	Discourage	Discourage
**HIV unknown**, high risk group or high risk region. Absence of mucosal risk factors	Recommended	Consider	Consider	Consider	Discourage	Discourage	Discourage
**HIV unknown**, no high risk group and no high risk region. Presence of mucosal risk factors	Consider	Consider	Consider	Consider	Discourage	Discourage	Discourage
**HIV unknown**, no high risk group and no high risk region. Absence of mucosal risk factors	Consider	Discourage	Discourage	Discourage	Discourage	Discourage	Discourage
**Sexual assault victim** (except in case of condom use or HIV negative test for aggressor)	Recommended	Recommended	Recommended	Recommended	Consider	Discourage	Discourage

**Source patient characteristics**	**Parenteral exposure**			
	Sharing syringe	Sharing other material (1)	Exposure to an abandoned syringe	Aggression with a used syringe
**HIV positive** or **unknown HIV status**	Recommended	Recommended	Consider if risk factors presents (2)	Consider if risk factors presents (2)

**Source patient characteristics**	**Mucosal exposure**		
	Bite	Corporal fluid exposure, non intact skin	Corporal fluid exposure, mucosal
**HIV positive or unknown**, high risk group or high risk region	Consider only if presence of blood inside aggressor mouth	Consider	Consider
**HIV unknown**, high risk group or high risk region	Discourage	Discourage	Discourage
