# Supplementary material for: Prescription of Non-Occupational Post-Exposure HIV Prophylaxis by Emergency Physicians: An Analysis on Accuracy of Prescription and Compliance
Source: PLoS One. 2016 Apr 12;11(4):e0153021. doi: 10.1371/journal.pone.0153021 (PMC4829160; doi:10.1371/journal.pone.0153021)
Supplement: S2 Table — (PDF) [file pone.0153021.s004.pdf]

Missing data for each variable of interest		
Variable	Missing data	%
Gender	0	0%
Compliance	0	0%
Country of origin (patient)	61	4%
Health insurance coverage	0	0%
Sexual orientation	0	0%
Age	0	0%
Multiple consultations for nPEP	0	0%
Sexual assault victim	0	0%
Type of exposure	0	0%
Number of partners	0	0%
Presence of mucosal lesions	285	21%
Source gender	19	1%
Source country of origin	154	11%
HIV status of the source	0	0%
Source being an IDU	556	41%
Source being a commercial sex worker	527	39%
Source being a former prisoner	558	41%
Source participating to orgies	551	41%
Source having multiple partners	510	38%
